# PtBiCoAgSn Multi-Component Alloy Electrocatalysts Enhancing the Oxidation of Ethylene Glycol to Value-Added C2 Products

**DOI:** 10.3390/molecules30193872

**Published:** 2025-09-24

**Authors:** Si-Tong Chen, Lin Wang, Hai-En Hou, Kang-Shuo Wang, Zhou Lan, Yao-Yue Yang, Wen-Bin Cai

**Affiliations:** 1 Key Laboratory of General Chemistry of the National Ethnic Affairs Commission, School of Chemistry and Environment, Southwest Minzu University, Chengdu 610041, China; c2570436346@163.com (S.-T.C.); wanglin1320310@163.com (L.W.); 19382084887@163.com (H.-E.H.); wks714805@outlook.com (K.-S.W.); lanzhou1440@163.com (Z.L.); 2 Shanghai Key Laboratory of Molecular Catalysis and Innovative Materials, Collaborative Innovation Center of Chemistry for Energy Materials, Department of Chemistry, Fudan University, Shanghai 200438, China

**Keywords:** multi-component alloys, platinum-based catalysts, ethylene glycol oxidation reaction, glycolic acid, C2 pathway

## Abstract

Ethylene glycol oxidation (EGOR) transforms waste plastic-derived chemicals into high-value products, representing an upcycling strategy that enhances resource efficiency. Pt-based electrocatalysts have shown promise for oxidizing ethylene glycol (EG) to high-value glycolic acid (GA), but they still suffer from high Pt usage, limited activity and stability, and poor low-potential selectivity. In this work, we report a highly dispersed PtBiCoAgSn multi-component alloy (MCA) electrocatalyst (denoted as MCA-PtBiCoAgSn) with outstanding catalytic activity and deactivation resistance, demonstrating a remarkable EGOR mass activity of 16.65 A ${\mathrm{mg}}_{\mathrm{Pt}}^{-1}$ at 0.76 V vs. RHE, which is 8-fold higher than that of commercial Pt/C (2.03 A ${\mathrm{mg}}_{\mathrm{Pt}}^{-1}$). Also, it can maintain an EGOR current density of 4.89 A ${\mathrm{mg}}_{\mathrm{Pt}}^{-1}$ after an extended long-term stability test. Additionally, it shows superior Faradaic efficiency (FE) for C2 products compared to Pt/C across the potential window of 0.5~0.9 V vs. RHE, with the FE of GA being up to 91% at a very low potential of 0.5 V vs. RHE. Moreover, in situ electrochemical infrared spectroscopy in a thin-layer configuration confirmed that EGOR proceeds via the C2 pathway on MCA-PtBiCoAgSn surfaces. This work may provide new insights into the design of high-efficiency and low-cost EGOR catalysts.

## 1. Introduction

With the dramatic increase in global plastic consumption, the recycling of waste plastics has become a pressing environmental issue and economic challenge that demands urgent solutions [1]. Poly(ethylene terephthalate) (PET), a major polyester material with global annual production exceeding 70 million metric tons, occupies a pivotal position in packaging and textile industries due to its exceptional mechanical properties and chemical stability [2]. The majority of surplus PET waste is either landfilled or discarded into natural environments, resulting in severe ecological pollution and significant resource depletion [3,4]. Consequently, depolymerizing PET into its monomers or small-molecule compounds (e.g., ethylene glycol, EG) creates new possibilities for the valorization of waste plastics [5].

As all known, EG serves as an important chemical feedstock; however, its characteristics of high volume and low price result in low added value, making it urgent to develop advanced technologies for its upgrading and utilization [6,7]. The electrocatalytic oxidation strategy undoubtedly represents a robust and reliable methodology [8,9,10]. Notably, electrocatalytic oxidation of EG into the high-value products aligns with the principles of green chemistry [11]. Nevertheless, the EG oxidation reaction (EGOR) exhibits intricate complexity, yielding a broad spectrum of products. These include C2 species such as glyoxylic acid (GLA), glyoxal, glycolic acid (GA), and oxalic acid (OA), as well as C1 products such as formic acid (FA) and CO_2_ [12]. Among them, GA and glycolate is high-valued and extensively utilized as the fine chemical and cleaning agent [6,11], which is one of the most desirable C2 products for EGOR. Generally, in the EGOR process, the C-C bond cleavage can lead to undesirable formic acid formation (C1 pathway). Thus, it requires precise control of C-C bond passivation and selective partial oxidation of C-H/C-O bonds to steer the reaction through the C2 pathway toward the target product GA [13,14]. Therefore, developing electrocatalysts with abundant reserves, low cost, and superior EGOR performance is critical to enable the economic feasibility of electrochemical upcycling for PET plastic waste [14,15,16].

Platinum-based materials have garnered significant attention in electrocatalysis due to their superior catalytic performance [17,18], and are widely recognized as the excellent catalysts for GA production [19]. However, the widespread adoption of Pt-based catalysts in electrocatalysis is hindered by inherent limitations, i.e., resource scarcity, high cost, structural instability, and rapid deactivation [20,21,22,23]. Typically, alloying is employed to address the aforementioned issues of Pt-based catalysts. However, although traditional binary alloys have been extensively studied, they are typically based on a single principal element (i.e., Pt). Their phase composition and microstructure are constrained by fixed eutectic points, eutectic ratios, and a limited variety of phases, resulting in a narrow window for catalysis tuning [24,25]. Moreover, these alloys often exhibit poor corrosion resistance in electrochemical environments and are prone to dealloying [26]. In contrast, the recent emergence of multicomponent alloys (MCAs)—especially high-entropy alloys (HEAs)—has introduced effects such as severe lattice distortion, sluggish diffusion, and multi-site synergy effect, leading to remarkable solid-solution strengthening and synergistic enhancement in overall performance [26,27]. Recently, several Pt-containing MCAs were reported to show significantly enhancement of the catalytic efficiency. Chen et al. [26] reported a multifunctional PtRhBiSnSb high-entropy intermetallic nanoplate catalyst that demonstrated remarkable mass activities for methanol, ethanol, and glycerol electrooxidation. Ao et al. [28] developed a multicomponent PdPtMoCrCoNi nanosheet catalyst that exhibits exceptional EGOR performance in alkaline media. Liu et al. [29] reported a Pt_1_-NiCoMgBiSn high-entropy alloy with single-atom Pt sites, which exhibits remarkable methanol oxidation activity of 35.3 A mg^−1^ and excellent CO anti-poisoning capability. Li et al. reported ultrasmall high-entropy alloy Pt_18_Ni_26_Fe_15_Co_14_Cu_27_/C nanoparticles, where the engineered surface of this catalyst facilitates rapid electron transfer for the oxidation of intermediates [30]. It is found that the multicomponent alloying can enable the incorporation of various non-precious metals to isolate contiguous Pt sites while harnessing the benefits of single-active-site catalysis [31]. Moreover, the intermetallic electronic modulation in MCAs can effectively weaken the adsorption strength of toxic intermediates [32,33]. Theoretical calculations revealed that the incorporation of metal atoms can enhance electron transfer efficiency, while the synergistic effects of oxophilic metals such as Bi, Sn, and Sb sites collectively stabilize the active centers [26]. Therefore, the development of Pt-based MCA electrocatalysts featuring ultralow Pt loading, superior overall performance and exceptional poisoning tolerance is of paramount importance for advancing EGOR applications.

In this work, we employ an impregnation–reduction synthesis method [34] to prepare a highly dispersed carbon-supported PtBiCoAgSn MCA catalysts (denoted as MCA-PtBiCoAgSn). With Pt as the primary active center responsible for EGOR catalytic activity, the introduction of secondary metals (Ag, Sn, Bi, Co) to form Pt alloys effectively disperses contiguous Pt-Pt bonds while accelerating the oxidation of C2 intermediates. The synergistic interaction among these elements yields exceptional improvements in EGOR activity, operational stability, GA selectivity. This study may provide an innovative Pt-based material solution for the valorization of EG and even the waste PET plastics.

## 2. Results and Discussion

### 2.1. Structure Characterization

**Figure 1:**
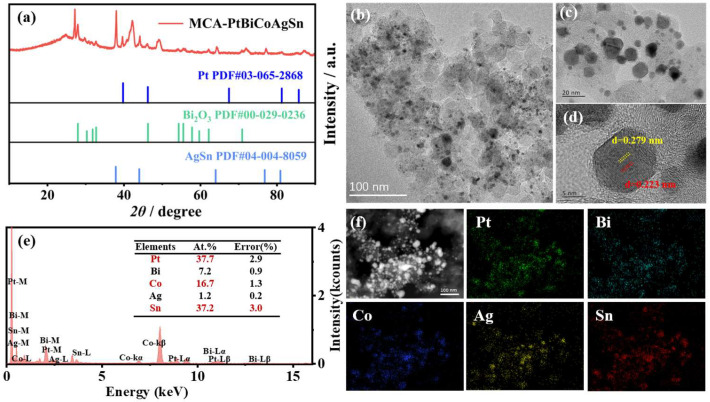
(**a**) X-ray diffraction (XRD) pattern of the as-prepared MCA-PtBiCoAgSn sample; (**b**,**c**) High-resolution transmission electron microscopy (HRTEM) images at different magnifications; (**d**) HRTEM image for one randomly selected nanoparticle from (**b**); (**e**) The quantitative composition analysis from the Energy dispersive X-ray spectroscopy (EDS); (**f**) EDS elemental mapping images.

By utilizing Pt as the central active site and incorporating non-precious metals (Bi, Co, Ag, Sn), the electronic structure of the catalyst can be precisely modulated, leading to optimized distribution of active sites and consequently a remarkable enhancement in catalytic performance. As shown in Figure 1a, the X-ray diffraction (XRD) pattern of MCA-PtBiCoAgSn reveals characteristic diffraction peaks of the Pt-based MCA phase are observed at 42.1°, 49.1°, 70.0°, and 87.1°, while it is also found that the AgSn alloy phase (37.9°, 44.1°, 64.2°, and 81.2°) and Bi_2_O_3_ phase could coexist in the as-prepared catalysts. This heterogeneous structure is not a synthetic defect but rather the key to its exceptional EGOR performance. Due to the differing electronegativities of Ag, Sn, and Pt, electron transfer occurs at the interfaces, modulating the d-band center of Pt [35]. Meanwhile, the Bi_2_O_3_ phase can transform into BiOOH under reaction potentials, generating abundant oxygen-containing species on its surface, the -OH species provided on its surface effectively oxidize and remove CO-like poisoning species from Pt sites [36,37,38]. The synergistic effect of these two factors significantly enhances the EGOR catalysis on the MCA-PtBiCoAgSn surface. In Figure 1b,c, high-resolution transmission electron microscopy (HRTEM) characterization at different magnifications reveals that the material consists of numerous nanoparticles. As shown in Figure S1, the average particle size of MCA-PtBiCoAgSn is 5.72 ± 2.93 nm and approximately Gaussian type distribution, indicating that our synthesis protocol still has room for improvement. Thus, the uniformity of the nanoparticles still needs to be improved. The lattice space in one randomly selected nanoparticle show apparently different size that can be 0.233~0.279 nm in Figure 1d, which can suggest that the as-prepared catalyst exhibits lattice mismatches and multiphase coexistence arising from the multi-element alloy composition. Simultaneously, Figure 1f presents the Energy dispersive X-ray spectroscopy (EDS) elemental mapping of MCA-PtBiCoAgSn, demonstrating homogeneous distribution of all five constituent elements (Pt, Bi, Co, Ag, Sn) at the nanoscale. The relative atomic percentages of constituent elements (Pt: 37.7%, Bi: 37.16%, Co: 7.16%, Ag: 16.67%, Sn: 1.23%) are quantitatively presented in the bar chart of Figure 1e, confirming the designed stoichiometry of the multi-metallic system. Table S1 quantitatively determines the bulk composition of MCA-PtBiCoAgSn (27.9 ± 0.23 wt.% total metal mass loading) through inductively coupled plasma optical emission spectrometry (ICP-OES), confirming the coexistence of all five metallic elements (Pt, Bi, Co, Ag, Sn) with atomic percentages of 20%, 19.1%, 32.6%, 22.8%, and 5.5%, respectively. The inherent differences in the physicochemical properties of the elements often cause the final composition of MCA-PtBiCoAgSn to deviate from the theoretical feeding ratio [34,39]. Preliminary evidence confirms that MCA-PtBiCoAgSn constitutes a well-dispersed multi-metallic alloy with non-negligible co-existing phases.

**Figure 2:**
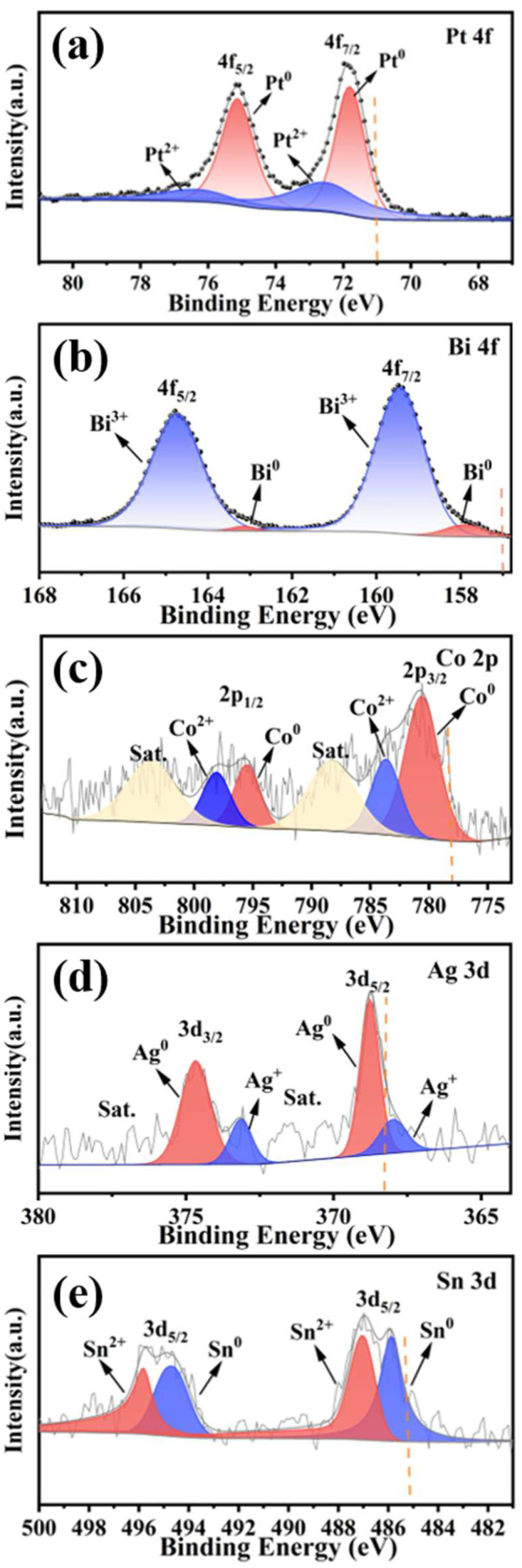
Core-level XPS spectra of as-prepared MCA-PtBiCoAgSn sample at the orbitals of (**a**) Pt 4f, (**b**) Bi 4f, (**c**) Co 2p, (**d**) Ag 3d, and (**e**) Sn 3d. The dash lines represent the standard value of the zero-valent metallic state of the corresponding metal.

Subsequently, X-ray photoelectron spectroscopy (XPS) analysis was employed to investigate the chemical states of constituent elements in MCA-PtBiCoAgSn, providing atomic-level insights into their electronic interactions. As shown in Figure 2a, Pt species exist in both Pt^2+^ and metallic Pt^0^ states. The binding energy of Pt^0^ 4f_7/2_ is measured at 71.78 eV, exhibiting a 0.78 eV positive shift compared to Pt^0^ (71.0 eV). As evidenced in Figure 2b, bismuth exists in both Bi^0^ and Bi^3+^ states, with Bi^3+^ being the dominant species. The Bi^0^ 4f_7/2_ peak appears at 157.90 eV, demonstrating a 0.90 eV positive binding energy shift relative to the standard value (157.00 eV) [40]. As presented in Figure 2c, the metallic Co in MCA-PtBiCoAgSn exhibits a binding energy of 780.62 eV for the Co 2p_3/2_ peak, demonstrating a significant 2.42 eV positive shift compared to the standard Co^0^ reference (778.2 eV). In Figure 2d, silver predominantly exists in the metallic Ag^0^ state and is highly dispersed across the catalyst surface. The Ag^0^ 3d_5/2_ peak appears at 368.77 eV, exhibiting a 0.57 eV positive binding energy shift relative to the standard Ag^0^ reference (368.2 eV). As revealed in Figure 2e, the Sn 3d spectrum exhibits characteristic spin–orbit splitting into 3d_3/2_ and 3d_5/2_doublet peaks. The peaks at 485.87 eV and 487.04 eV correspond to the Sn 3d_5/2_ level, where the 485.87 eV binding energy represents metallic Sn^0^ demonstrating a 0.67 eV positive shift relative to the standard Sn^0^ reference (485.2 eV). Consequently, comprehensive analysis of binding energies and valence states across all elements confirms significant orbital-specific binding energy shifts, unequivocally demonstrating the existence of electronic interplay between Pt and adjacent metals in the alloy system. Such inter-component electronic interactions will positively modulate both the activity and selectivity of EGOR, as detailed below.

### 2.2. EGOR Performance

**Figure 3:**
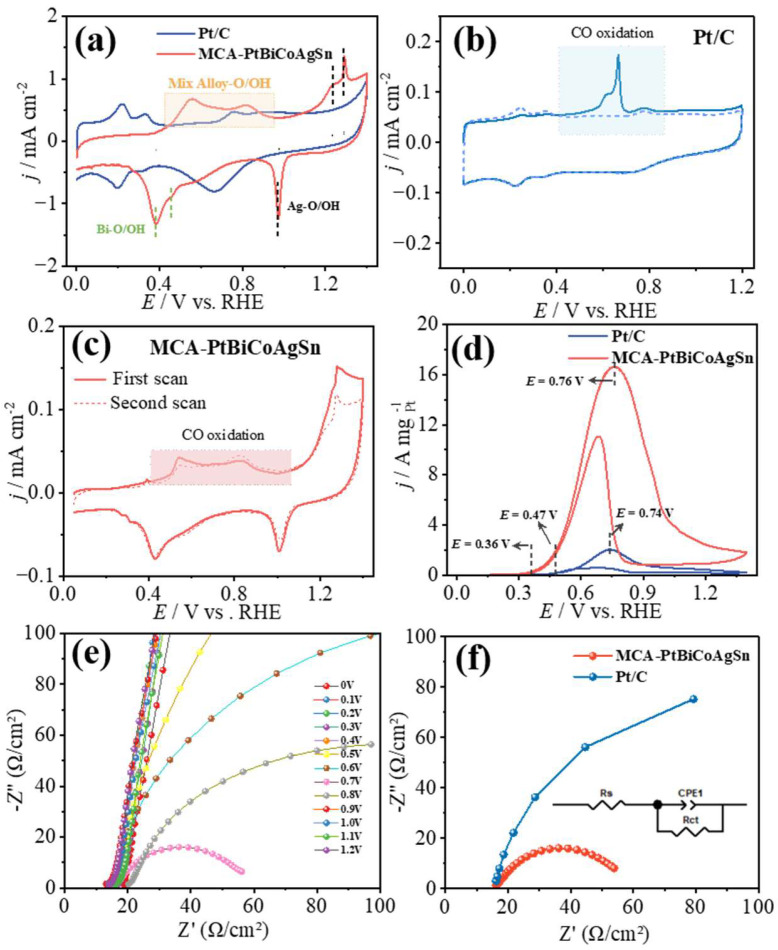
(**a**) CV curves of MCA-PtBiCoAgSn and Pt/C catalysts in 1 M KOH electrolyte at a scan rate of 50 mV s^−1^; CO stripping voltammograms in 1 M KOH at (**b**) Pt/C and (**c**) MCA-PtBiCoAgSn; (**d**) CV curves of MCA-PtBiCoAgSn and Pt/C in 1 M KOH + 1 M EG solution at 50 mV s^−1^; (**e**) Nyquist plots on MCA-PtBiCoAgSn measured at 0-1.2 V vs. RHE in Ar-saturated 1 M EG + 1 M KOH solution.; (**f**) Nyquist plots of electrochemical impedance spectroscopy (EIS) for the EGOR on MCA-PtBiCoAgSn and Pt/C catalysts at 0.7 V vs. RHE, the inset shows the simplified equivalent circuit used for EIS fitting.

As shown in Figure 3a and Figures S2–S5, the electrochemical behaviors of MCA-PtBiCoAgSn, Pt/C, Bi/C, Co/C, Ag/C, and Sn/C catalysts were systematically evaluated by cyclic voltammetry (CV) in 1 M KOH electrolyte. First, distinct differences in the hydrogen adsorption/desorption potential window are observed between the CV profiles of MCA-PtBiCoAgSn and control samples (Pt/C, Co/C, Sn/C), clearly demonstrating that the contiguous Pt sites are disrupted by the intervening foreign-metal components. The pronounced current fluctuations within the 0.45–0.95 V vs. RHE originate from the competitive adsorption/desorption of O* and OH* species on the MCA-PtBiCoAgSn surface. The incorporation of Bi and Ag dopants in MCA-PtBiCoAgSn is evidenced by distinct O*/OH* adsorption features in its CV profile, corresponding to oxophilic Bi sites and surface-oxidized Ag species.

Meanwhile, CO molecules were employed as the probe to show the surface structure difference between MCA-PtBiCoAgSn and Pt/C. CO stripping voltammograms in 1 M KOH solution is presented in Figure 3b,c and Figures S6–S9, a distinct CO oxidation peak emerges at 0.66 V vs. RHE for Pt/C due to CO surface adsorption, whereas Bi/C, Co/C, Ag/C, and Sn/C catalysts exhibit negligible CO oxidation activity. Thus, the incorporation of non-precious metals (Bi, Co, Ag, Sn) into MCA-PtBiCoAgSn significantly suppresses CO adsorption on the catalyst surface. Thus, the formation of a multicomponent alloy not only disrupts contiguous Pt sites but also suppresses the adsorption of surface species through the incorporation of oxophilic elements. Such structural modulation plays a pivotal role in enabling EG to follow the C2 pathway and undergo rapid oxidation on the surface, thereby enhancing the selectivity toward the desired C2 products (vide infra). It should be acknowledged that the significant oxidation current observed in the high potential region (E > 1.2 V) may be attributed to the possible anodic oxidation and even dissolution (if present) of non-noble metals under such high potentials. This process leads to surface reconstruction, ultimately resulting in substantially higher irreversible oxidation currents in this region. These findings demonstrate that our multi-principal-element catalyst does not form a single solid-solution phase. As clearly shown by the XRD data (Figure 1a), it inevitably contains mono- or multi-metallic segregated phases. This conclusion is further corroborated by the significant metal leaching observed during prolonged electrolysis (Table S2).

On the other hand, as shown in Figure 3d and Figures S2–S5, the electrocatalytic performance of MCA-PtBiCoAgSn, Pt/C, Bi/C, Co/C, Ag/C, and Sn/C catalysts was systematically evaluated in 1 M KOH + 1 M EG. Bi/C, Co/C, Ag/C, and Sn/C catalysts exhibit negligible EGOR activity. The onset oxidation potential serves as a critical parameter for evaluating catalyst activity [41,42], where a lower potential indicates superior catalytic performance in initiating the reaction. During the anodic scan, the onset potential of MCA-PtBiCoAgSn (0.36 V vs. RHE) is significantly lower than that of Pt/C (0.47 V vs. RHE), demonstrating a 110 mV reduction in activation potential. The prominent oxidation peak observed at 0.76 V vs. RHE unequivocally corresponds to ethylene glycol oxidation on MCA-PtBiCoAgSn surfaces. At this peak potential, the catalyst achieves an exceptional EGOR mass activity of 16.65 A ${\mathrm{mg}}_{\mathrm{Pt}}^{-1}$, surpassing that of as-prepared Pt/C (2.03 A ${\mathrm{mg}}_{\mathrm{Pt}}^{-1}$) and that of the commercial Pt/C [43,44]. Meanwhile, its EGOR mass activity is significantly superior to that of other recently reported Pt-based catalysts, as shown in Table 1. This may be attributed to the synergistic effect between metal atoms, which can expose more active sites, accelerate the oxidation of ethylene glycol, and facilitate the conversion of oxidation intermediates.

This indicates faster reaction kinetics on the MCA-PtBiCoAgSn surface, demonstrating its superior capability for rapid EGOR [45,46], consistent with the aforementioned results. Furthermore, we conducted electrochemical impedance spectroscopy (EIS) measurements of the catalysts in 1 M KOH + 1 M EG solution [47]. As shown in Figure 3e, significant surface electron transfer behavior was observed for MCA-PtBiCoAgSn at various potentials (0–1.2 V vs. RHE) in Ar-saturated 1 M KOH + 1 M EG solution. Notably, the charge transfer resistance (*R*ct) reached its minimum value of 40.99 Ω at 0.7 V vs. RHE, which is significantly lower than that of Pt/C (152.1 Ω) under identical conditions, as further illustrated in Figure 3f.

**Figure 4:**
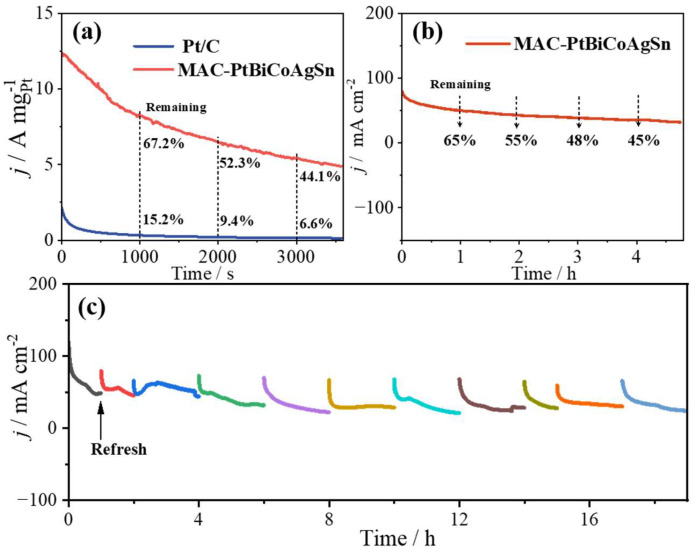
(**a**) *i*-t curves collected at MCA-PtBiCoAgSn and Pt/C catalysts in 1 M KOH + 1 M EG solution at 0.7 V vs. RHE for 3600 s; (**b**) Long-term *i*-t curve collected at MCA-PtBiCoAgSn in 1 M KOH + 1 M EG solution with the applied potential of 0.7 V vs. RHE by depositing the catalyst ink onto the carbon paper as the working electrode; (**c**) multiple stability tests at MCA-PtBiCoAgSn catalyst with a total duration of 19 h.

Subsequently, chronoamperometry (CA) measurements were conducted to evaluate the stability of MCA-PtBiCoAgSn in 1 M KOH + 1 M EG solution, with all tests performed at a constant potential of 0.7 V vs. RHE. As shown in Figure 4a, MCA-PtBiCoAgSn demonstrates significantly superior EGOR stability compared to Pt/C. At testing durations of 1000 s, 2000 s, and 3000 s, MCA-PtBiCoAgSn retains 67%, 52%, and 44.1% of its initial activity, respectively, all substantially higher than Pt/C. After 3600 s of continuous operation, MCA-PtBiCoAgSn maintains a considerable current density of 4.89 A ${\mathrm{mg}}_{\mathrm{Pt}}^{-1}$, while Pt/C exhibits nearly complete activity loss (approaching 0 A ${\mathrm{mg}}_{\mathrm{Pt}}^{-1}$). In addition, we further evaluated the long-term EGOR stability of MCA-PtBiCoAgSn by depositing the catalyst ink onto the carbon paper for extended testing periods up to 4 h for one time and a total of 19 h across multiple tests, as demonstrated in Figure 4b and Figure 4c, respectively. Notably, MCA-PtBiCoAgSn maintains remarkable current density retention of 65%, 55%, 48%, and 45% at 1 h, 2 h, 3 h, and 4 h intervals, respectively. To determine whether the composition of MCA-PtBiCoAgSn changed after prolonged electrolysis for 4 h, we performed an extended electrolysis test in 1 M KOH + 1 M EG solution. Throughout this process, the MCA-PtBiCoAgSn catalyst exhibited significant elemental leaching and surface reconstruction. As shown in Table S2, the atomic ratios of metallic elements shifted from Pt:Bi:Co:Ag:Sn = 19.8:19.1:32.6:22.8:5.5 before electrolysis to Pt:Bi:Co:Ag:Sn = 25.77:9.52:27.24:20.47:16.80 after electrolysis. multi-principal-element catalyst is not a single sol-id-solution phase; instead, as the XRD data (Figure 1a) clearly show, it inevitably contains mono- or mul-ti-metallic segregated phases [26,31,32]. Furthermore, extended CA testing reveals that the catalyst exhibits excellent regenerative capability, with stable current recovery observed after each electrolyte replenishment. Therefore, compared with the literature (Table 1), we can state that this as-prepared catalyst exhibits EGOR catalytic stability comparable to the best-reported catalysts to date.

**Table 1:** Comparison of EGOR performance between MCA-PtBiCoAgSn and previously reported Pt-based catalysts.

Catalysts	Mass Activity	Stability	*FE*_GA_	Ref.
MCA-PtBiCoAgSn	16.65 A ${mg}_{Pt}^{-1}$	65% (1 h)	91%	This work
(PtIr) (FeMoBi)	5.2 A ${mg}_{Pt}^{-1}$	66.7% (1 h)	95%	[43]
PdPtMoCrCoNi	10.12 A ${mg}_{Pt+\mathrm{Pd}}^{-1}$	50% (5000 s)	/	[28]
PtBiNiCoSn/C	1.406 A ${mg}_{Pt}^{-1}$	46.5% (3000 s)	/	[44]
PtPdAuNiCo/C	0.482 A ${mg}_{Pt+\mathrm{Pd}+\mathrm{Au}}^{-1}$	18% (3000 s)	/	[48]
Ir-PdPtAuNiCu	2.41 A ${mg}_{Pt+\mathrm{Pd}}^{-1}$	89% (20 h)	88.8%	[49]
PtRh	4.677 A ${mg}_{Pt}^{-1}$	12% (1 h)	/	[50]
Pt/*γ*-NiOOH	2.532 A ${mg}_{Pt}^{-1}$	/	85%	[51]

### 2.3. Product Distribution of EGOR

**Figure 5:**
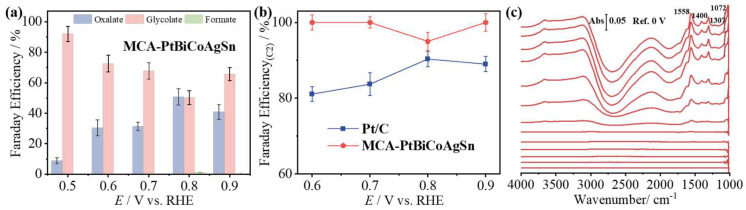
(**a**) *FE*s of various products during EG oxidation on MCA-PtBiCoAgSn in 1 M KOH + 1 M EG solution (0.5–0.9 V vs. RHE); (**b**) Comparison of the *FE* of C2 products obtained at MCA-PtBiCoAgSn and Pt/C catalysts. (**c**) in situ electrochemical IRA spectra collected at MCA-PtBiCoAgSn electrode in 1 M KOH + 1 M EG solution with a scan rate of 5 mV s^−1^, using the single-beam spectrum at 0 V vs. RHE as the reference spectrum.

To investigate the product distribution of EG electrolysis on MCA-PtBiCoAgSn during the EGOR process, high-performance liquid chromatography (HPLC) analysis was employed. Additionally, the concentrations of various products were quantified using the external standard calibration method, with corresponding calibration curves presented in Figure S10. As shown in Figure 5a, C2 products dominate across the 0.5–0.9 V vs. RHE potential window, achieving a Faradaic efficiency of GA (*FE*_GA_) up to 91% at a very low potential of 0.5 V vs. RHE. In contrast, Pt/C catalysts predominantly generate formic acid (FA) as a byproduct under identical conditions (Figure S11). As demonstrated in Figure 5b, the Faradaic efficiency for C2 products (*FE*_C2_) of MCA-PtBiCoAgSn significantly surpasses that of Pt/C across the 0.6–0.9 V vs. RHE potential range. The multi-component alloying strategy in MCA-PtBiCoAgSn effectively steers the EGOR pathway toward preferential C2 product formation. In situ electrochemical infrared absorption spectroscopy (IRAS) in the thin-layer configuration [52,53,54,55] collected during EGOR on the MCA-PtBiCoAgSn surface, as shown in Figure 5c, show the characteristic absorption peaks at 1558, 1400, 1307, and 1072 cm^−1^ those can be assigned to glycolate species. This further confirms that the EGOR on the MCA-PtBiCoAgSn surface follows the C2 pathway [56,57]. This result demonstrates that the multi-component doping (Pt-Bi-Co-Ag-Sn) effectively modulates the reaction pathway on the catalytic surface, steering the EGOR process toward preferential formation of C2 products.

## 3. Materials and Methods

### 3.1. Chemicals and Materials

Platinum (II) chloride (PtCl_2_, 10025-65-7), tin (II) chloride dihydrate (SnCl_2_·2H_2_O, AR, 10025-65-1), silver nitrate (AgNO_3_, AR, 7761-88-8), cobalt (II) chloride hexahydrate (CoCl_2_·6H_2_O, AR grade, 7791-13-1), bismuth (III) nitrate pentahydrate (Bi(NO_2_)_2_·5H_2_O, 10035-06-0), hydrochloric acid (HCl, GR, 7647-01-0), and ethylene glycol (EG, 107-21-1, 99.5%) were purchased from Chengdu Kelong Chemical Co., Ltd. (Chengdu, China). Activated carbon powder (Vulcan XC72) was obtained from Cabot Chemical Co., Ltd. (Shanghai, China). The Nafion perfluorinated resin solution was purchased from Adamas Reagent Co., Ltd. (Shanghai, China) High-purity Ar (99.99%) and the 5% H_2_/Ar mixed gas were obtained from Sichuan Qiaoyuan Gas Co., Ltd. (Chengdu, China). Other chemicals and reagents consisted of ultrapure water (18.25 MΩ·cm). All chemical reagents were used as received without further purification.

### 3.2. Synthesis of Catalysts

**Preparation of precursor powder.** First, 100 mg of a carbon-supported catalyst with Pt, Ag, Sn, Co, and Bi feeding ratio of 1:1 (10 wt.% Pt loading) was prepared in a beaker. At room temperature, 5 mL of deionized water and 1.2 mL of hydrochloric acid (11.639 mol L^−1^) were added, followed by ultrasonication for 60 min. Subsequently, the mixture was stirred and dried in a water bath at 70 °C using a Heidolph magnetic stirrer (Shanghai, China), and then dried overnight in a vacuum oven at 60 °C.

**Synthesis of carbon-supported PtBiCoAgSn Catalysts.** First, the overnight-dried precursor powder was thoroughly ground and placed in a clean quartz boat. It was then transferred into a tube furnace, and high-purity Ar was introduced for approximately 30 min to purge the air inside the tube. Finally, a 5% H_2_/Ar mixed gas was introduced, and the sample was calcined at 650 °C for 1 h with a heating rate of 10 °C min^−1^. After cooling to room temperature, the PtBiCoAgSn powder was collected.

**Preparation of other carbon-supported metal catalysts.** Each monometallic carbon-supported catalysts (denoted as Pt/C, Ag/C, Sn/C, Co/C, and Bi/C) were prepared using the sodium borohydride reduction method [58,59]. 20 mg of the catalyst (10 wt.% loading) was prepared by adding 5 mL of deionized water and sonicating for 60 min to ensure uniform dispersion. Under an ice-water bath, a sodium borohydride solution (prepared by dissolving 19.7 mg NaBH_4_ and 35.15 mg Na_2_CO_3_ in 10 mL deionized water) was slowly added via a peristaltic pump at a rate of 6 rpm while stirring on a Heidolph magnetic stirrer. After complete addition of the NaBH_4_ solution, the mixture was heated to 70 °C in a water bath until fully dried, followed by overnight vacuum drying at 60 °C.

### 3.3. Materials Characterization

The morphology and microstructure of MCA-PtBiCoAgSn were characterized using high-resolution aberration-corrected scanning transmission electron microscopy (HR-TEM) bright-field images and elemental mapping were performed on the MCA-PtBiCoAgSn using a JEOL F200 instrument (Akishima, Japan). The crystalline phase of the samples was characterized by X-ray diffraction (XRD, Malvern Panalytical X’Pert3 Powder, Almelo, The Netherlands). The actual metal content in the samples was determined using inductively coupled plasma optical emission spectrometry (ICP-OES, Agilent-5110, Santa Clara, CA, USA). X-ray photoelectron spectroscopy (XPS) analysis was performed using a Thermo Scientific K-Alpha spectrometer (Waltham, MA, USA), and all the XPS spectra were calibrated using the C 1s peak.

### 3.4. Electrochemical Measurements

Electrochemical measurements were performed at room temperature using a CHI 660 electrochemical workstation (three-electrode system). The catalyst ink was drop-cast onto a glassy carbon electrode (3 mm in diameter, 0.07 cm^2^ geometric area) serving as the working electrode (WE), with a graphite rod as the counter electrode (CE)and a saturated calomel electrode (SCE) as the reference electrode. All potentials in this study are reported versus SCE and were converted to the reversible hydrogen electrode (RHE) scale according to Equation (1):

(1) E(vs. RHE) = E(vs. SCE) + 0.059 × pH + 0.242V

The electrocatalytic activities of MCA-PtBiCoAgSn and Pt/C catalysts were evaluated through the EGOR. Typically, 2 mg of the electrocatalyst was ultrasonically dispersed in a mixture containing 500 μL ethanol, 500 μL H_2_O, and 120 μL Nafion solution (5 wt.%) to form a homogeneous ink. Subsequently, 5.6 μL of the catalyst ink was drop-cast onto a glassy carbon electrode (GCE, *Φ* = 3 mm) surface and dried at room temperature. All electrocatalysts were characterized by cyclic voltammetry (CV) in Ar-saturated 1 M KOH solution at a scan rate of 50 mV s^−1^. The electrocatalytic activity for EGOR was evaluated in Ar-saturated 1 M KOH + 1 M EG solution using the same scan rate of 50 mV s^−1^. The stability of the catalysts was evaluated by chronoamperometry (i-t) measurements. Specifically, 22.4 μL of the catalyst ink was drop-cast onto a glassy carbon electrode (GCE, *Φ* = 6 mm) surface and dried at room temperature. The catalyst ink was similarly drop-cast onto carbon paper (2 × 2 cm^2^) for long-termstability testing. Electrochemical impedance spectroscopy (EIS) measurements were conducted in 1 M KOH + 1 M EG electrolyte solution under various applied potentials (0~1.2 V vs. RHE), with an amplitude of 5 mV and a frequency sweep ranging from 0.0464159 Hz to 100 kHz.

### 3.5. CO Stripping

The CO stripping experiments were performed in Ar-saturated 1 M KOH solution using potentiostatic coulometry (BE). Specifically, CO gas was purged into the solution for approximately 30 min at a constant potential of 0.05 V vs. RHE, followed by Ar purging for about 40 min to achieve CO saturation adsorption on the electrode surface. Subsequently, the CO stripping voltammograms were obtained by CV at a scan rate of 5 mV s^−1^ to characterize the CO oxidation behavior on the catalyst surface.

### 3.6. Products Analysis

The EGOR products were quantitatively analyzed using an LC-5090 high-performance liquid chromatography (HPLC) system (Fuli Instruments Co., Ltd., Wenling, China). The chromatographic system was equipped with a Shimadzu GWS ChromCore Sugar-10H analytical column (Kyoto, Japan, 7.8 mm × 300 mm, particle size: 6 μm) and a UV-Vis detector set at 210 nm. The chromatographic conditions were as follows: mobile phase of 25 mmol/L H_2_SO_4_ aqueous solution, column temperature maintained at 35 °C, and flow rate set at 1.0 mL/min.

During the electrochemical oxidation experiments, precisely 22.4 μL of electrolyte at the working electrode interface was collected using a micropipette under various applied potential conditions. The samples were immediately quenched with 0.5 M H_2_SO_4_ solution to terminate the reaction. The dilution factor was dynamically adjusted (5–20 fold gradient dilution) based on real-time monitoring of the electrolytic charge quantity (*Q*). The final solution was filtered through a 0.22 μm microporous membrane prior to injection for analysis. The diluted samples were then analyzed by HPLC, where oxidation products were quantified using UV-Vis detection. Prior to analysis, external calibration curves were established for all potential reaction products across relevant concentration ranges (see Supplementary Materials). This enabled quantification of individual EGOR products and subsequent calculation of FE using Equation (2) [19]:

(2) $$FE=\frac{C\;\times \;V\;\times \;n\;\times \;F}{Q_{\mathrm{total}}}$$

where *C* corresponds to the concentration of products quantified by HPLC analysis, *V* represents the volume of the sampled electrolyte solution, *Q*_total_ represents the total charge passed during the electrolysis process, while *F* denotes the Faraday constant (96,485 C mol^−1^). The parameter *n* represents the number of electrons transferred during the formation of each product. The production of Glycolic acid, Oxalic acid, and Formic acid products requires the oxidation process that involve the transfer of 4, 8, and 3 electrons, respectively.

### 3.7. In Situ Electrochemical Infrared Absorption Spectral (IRAS) Measurements

The electrochemical EGOR process was monitored in real time using in situ electrochemical infrared absorption spectroscopy (IRAS) in external reflection mode, with detailed experimental procedures available in elsewhere [19,52,53]. The experimental procedure was conducted as follows: A glassy carbon electrode (*Φ* = 6 mm) modified with catalyst ink served as the WE, while a SCE and platinum foil functioned as the reference electrode and CE, respectively. These three electrodes were integrated into a custom-designed infrared spectroelectrochemical cell to establish a three-electrode system.

Prior to measurements, the spectrometer sample compartment was rigorously purged with high-purity nitrogen gas (N_2_, purity ≥ 99.999%) until complete removal of residual CO_2_ and water vapor was achieved. Throughout the testing process, a continuous N_2_ flow (0.5 L/min) was maintained as a protective atmosphere to prevent atmospheric interference. Subsequently, the WE was precisely coupled with a CaF_2_ prism to form an ultrathin electrolyte layer approximately 10 μm thick. Infrared spectral intensity was quantified using absorbance (denoted as *A* or *Abs*.), defined as −log(*I*/*I*_0_), where *I*_0_ and *I* represent the infrared radiation intensity under reference and sample conditions, respectively.

During EGOR measurements in external reflection mode, an unpolarized infrared beam was incident at 55° to undergo total internal reflection at the prism interface. The beam subsequently penetrated the thin electrolyte layer and was reflected at the WE surface, before being ultimately detected by a liquid-nitrogen-cooled mercury cadmium telluride (MCT) infrared detector.

## 4. Conclusions

This study employed the impregnation–reduction method to synthesize a highly dispersed multi-component alloy composed of Pt, Bi, Co, Ag, and Sn. The alloy can independently leverage the catalytic advantages of single active sites, exhibiting high EGOR activity, exceptional stability, superior selectivity for C2 products, and outstanding CO anti-poisoning capability. STEM-EDS revealed the uniform distribution of constituent elements, while XRD, XPS, and electrochemical performance tests were employed to thoroughly investigate the structure-activity relationship of the MCA-PtBiCoAgSn alloy. The synergistic interaction of the five constituent atoms—enabled by their distinct atomic sizes and electronegativities—led to the formation of MCA-PtBiCoAgSn with a unique structure, which exhibited superior electrochemical performance. Notably, the MCA-PtBiCoAgSn catalyst demonstrated an exceptional EGOR mass activity of 16.65 A ${mg}_{Pt}^{-1}$ at 0.76 V vs. RHE—an eightfold enhancement compared to conventional Pt/C catalysts. HPLC analysis of the EGOR products confirmed that the *FE*_C2_ consistently exceeded 90% on the MCA-PtBiCoAgSn surface. Therefore, the MCA-PtBiCoAgSn catalyst designed in this work represents a highly active, stable, and selective electrocatalyst that effectively reduces costs while opening new possibilities for the value-added utilization of organic small molecules.

## Data Availability

The original contributions presented in the study are included in the article. Further inquiries can be directed to the corresponding author.

## Supplementary Materials

The following supporting information can be downloaded at: https://www.mdpi.com/article/10.3390/molecules30193872/s1, Figure S1: Average particle size of the as-prepared MCA-PtBiCoAgSn sample, it was obtained by measuring the diameters of 500 randomly selected nanoparticles from the HRTEM images; Figure S2: The CV curves of Bi/C in 1 M KOH and 1 M KOH + 1M EG solution at a scan rate of 50 mV s^−1^; Figure S3: The CV curves of Co/C in 1 M KOH and 1 M KOH + 1M EG solution at a scan rate of 50 mV s^−1^; Figure S4: The CV curves of Ag/C in 1 M KOH and 1 M KOH + 1M EG solution at a scan rate of 50 mV s^−1^; Figure S5: The CV curves of Sn/C in 1 M KOH and 1 M KOH + 1M EG solution at a scan rate of 50 mV s^−1^; Figure S6: The CO stripping curves of Bi/C in 1 M KOH electrolyte at a scan rate of 50 mV s^−1^; Figure S7: The CO stripping curves of Co/C in 1 M KOH electrolyte at a scan rate of 50 mV s^−1^; Figure S8: The CO stripping curves of Ag/C in 1 M KOH electrolyte at a scan rate of 50 mV s^−1^; Figure S9: The CO stripping curves of Sn/C in 1 M KOH electrolyte at a scan rate of 50 mV s^−1^; Figure S10: The high-performance liquid chromatography (HPLC) calibration curves of (a) Glycolic acid, (b) Oxalic acid, (c) Formic acid, and (d) Glyoxylic acid from 1 ppm to 200 ppm; Figure S11: The Faradaic efficiency (*FE*) of various products on Pt/C in 1 M KOH and 1 M EG solution within the potential range of 0.5–0.9 V vs. RHE; Table S1: The ICP-OES analysis results of five constituent elements of the as-prepared MCA-PtBiCoAgSn; Table S2: The ICP-OES analysis results of five metallic elements before and after 4-h electrolysis in 1 M KOH + 1 M EG solution.
