# Supplementary material for: PtBiCoAgSn Multi-Component Alloy Electrocatalysts Enhancing the Oxidation of Ethylene Glycol to Value-Added C2 Products
Source: Molecules. 2025 Sep 24;30(19):3872. doi: 10.3390/molecules30193872 (PMC12526256; doi:10.3390/molecules30193872)
Supplement: Supplementary file 1 [file molecules-30-03872-s001.zip › molecules-3816594-supplementary.pdf]

PtBiCoAgSn Multi-Component Alloy Electrocatalysts Enhancing the Oxidation of Ethylene Glycol to Value-Added C2 Products

Si-Tong Chen [1,†], Lin Wang [1,†], Hai-En Hou [1], Kang-Shuo Wang [1], Zhou Lan [1], Yao-Yue Yang [1,*] and Wen-Bin Cai [2,*]

1 Key Laboratory of General Chemistry of the National Ethnic Affairs Commission, School of Chemistry and Environment, Southwest Minzu University, Chengdu 610041, China; c2570436346@163.com (S.-T.C.); wanglin1320310@163.com (L.W.); 19382084887@163.com (H.-E.H.); wks714805@outlook.com (K.-S.W.); lanzhou1440@163.com (Z.L.)

2 Shanghai Key Laboratory of Molecular Catalysis and Innovative Materials, Collaborative Innovation Center of Chemistry for Energy Materials, Department of Chemistry, Fudan University, Shanghai 200438, China

* Correspondence: yaoyueyoung@swun.edu.cn (Y.-Y.Y.); wbcai@fudan.edu.cn (W.-B.C.)

† These authors contributed equally to this work.

Table S1. The ICP-OES results of five constituent elements of the as-prepared MCA-PtBiCoAgSn.

Element	Atom ratio(%)	Error(%)
Pt	19.8	0.11
Bi	19.1	0.33
Co	32.5	0.18
Ag	22.8	0.03
Sn	5.8	0.0045

Table S2. The ICP-OES results of five metallic elements before and after 4-hour electrolysis in 1 M KOH + 1 M EG solution.

**Element**	**Before electrolysis (at.%)**	**After electrolysis (at.%)**
Pt	19.8	25.77
Bi	19.1	9.52
Co	32.6	27.42
Ag	22.8	20.47
Sn	5.5	16.80

Analysis of the electrolyte solution revealed dissolved metal concentrations of 69.23367 μg/L for Pt, 183.14133 μg/L for Bi, 194.16633 μg/L for Co, 72.22417 μg/L for Ag, and 215.39533 μg/L for Sn.

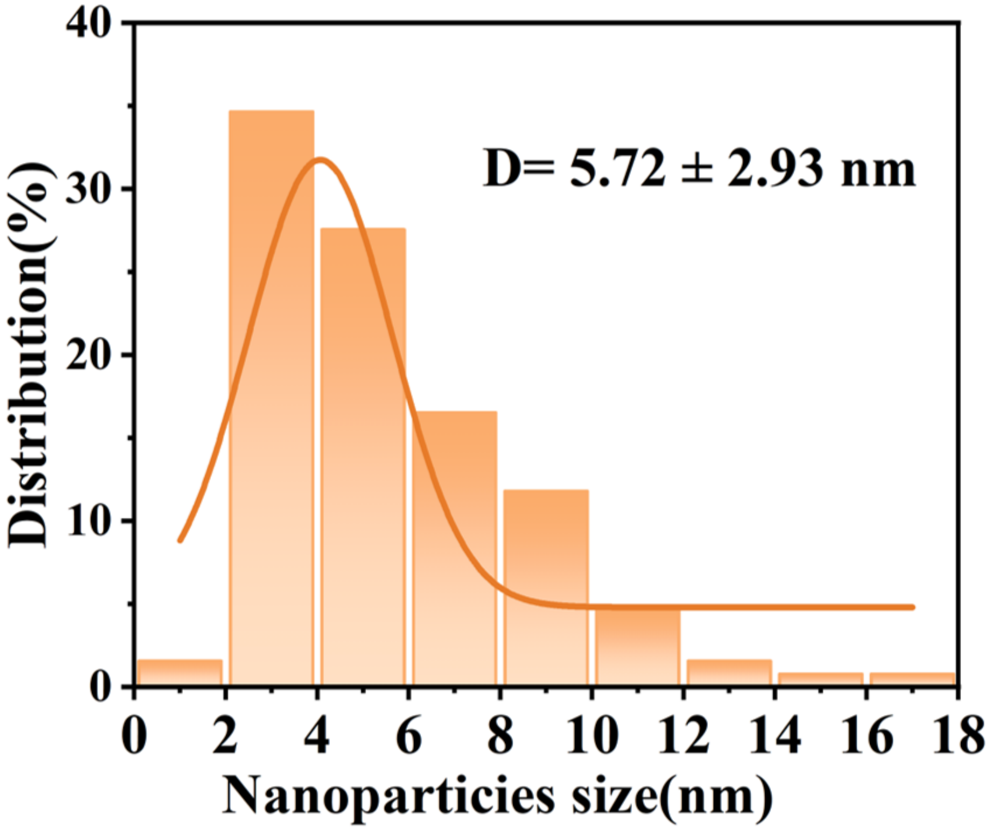

Figure S1. Average particle size of the as-prepared MCA-PtBiCoAgSn sample, it was obtained by measuring the diameters of 500 randomly selected nanoparticles from the HRTEM images.

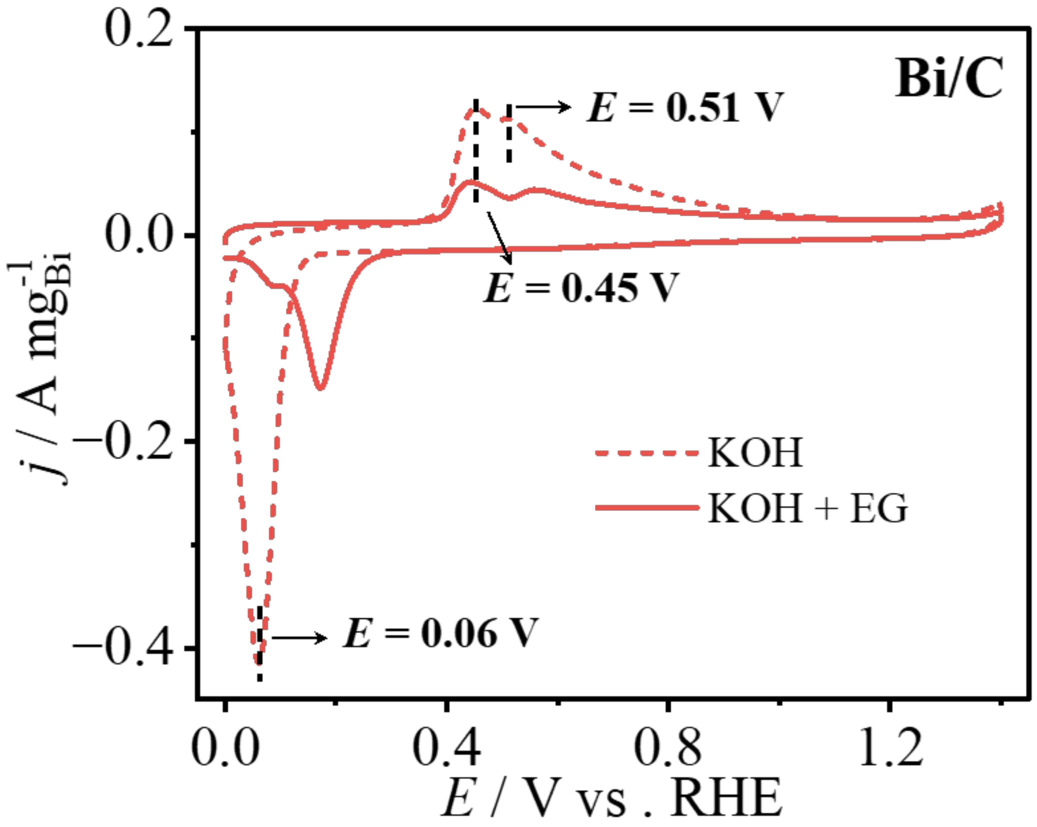

Figure S2. The CV curves of Bi/C in 1 M KOH and 1 M KOH + 1M EG solution at a scan rate of 50 mV s^{-1}.

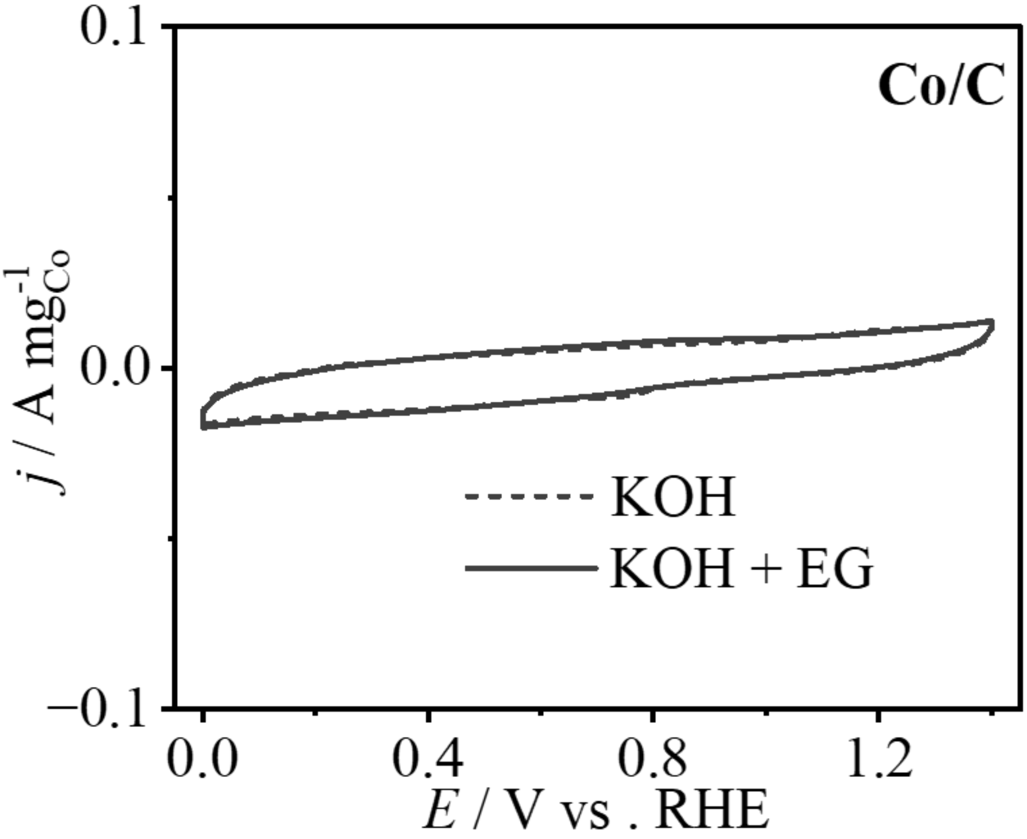

Figure S3. The CV curves of Co/C in 1 M KOH and 1 M KOH + 1M EG solution at a scan rate of 50 mV s^{-1}..

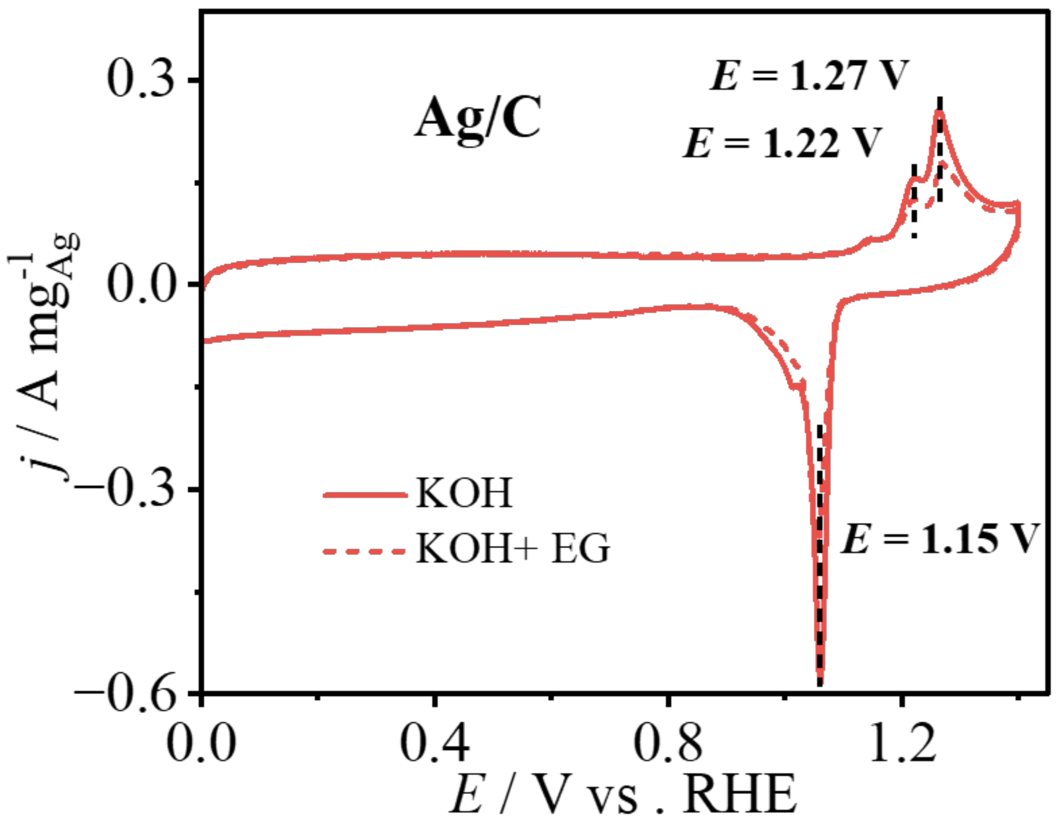

Figure S4. The CV curves of Ag/C in 1 M KOH and 1 M KOH + 1M EG solution at a scan rate of 50 mV s^{-1}.

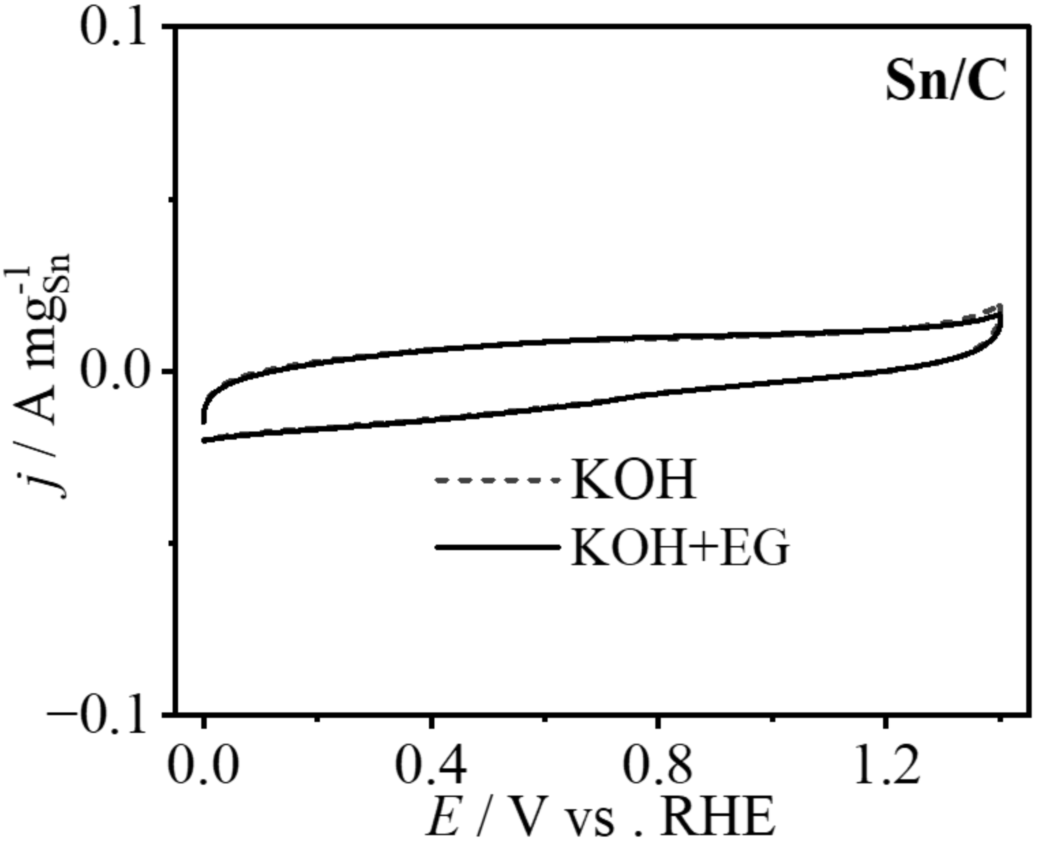

Figure S5. The CV curves of Sn/C in 1 M KOH and 1 M KOH + 1M EG solution at a scan rate of 50 mV s^{-1}.

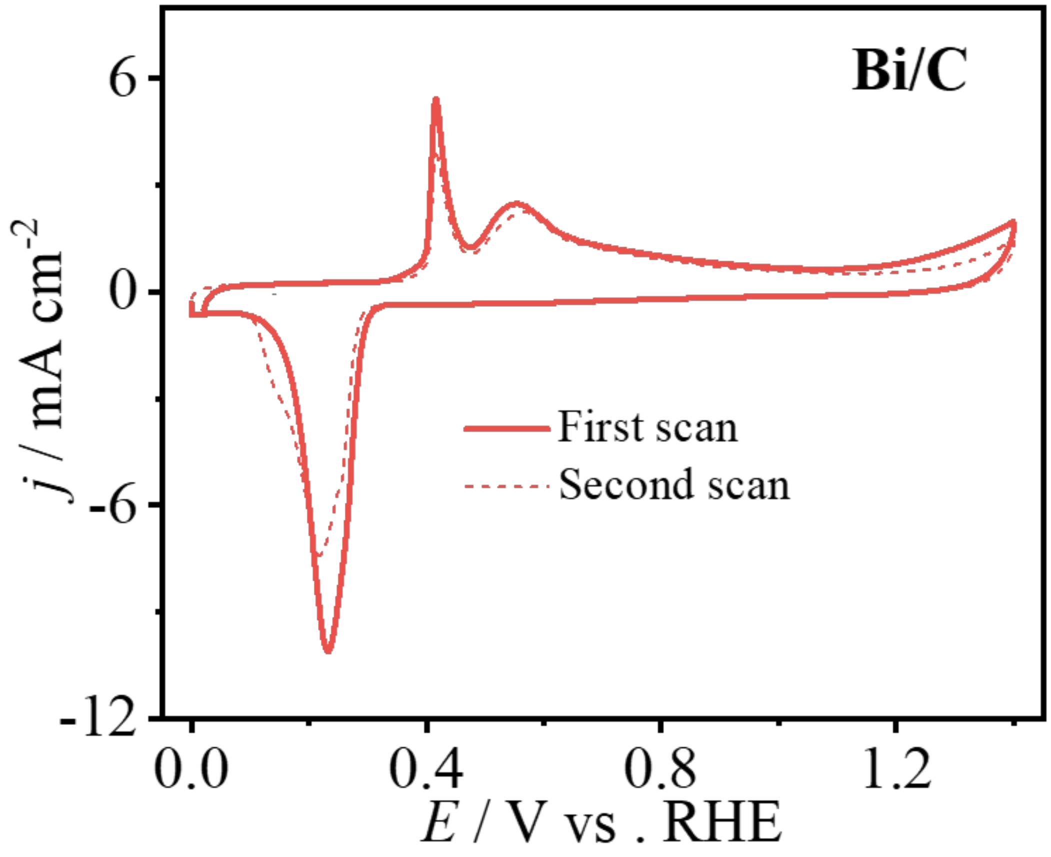

Figure S6. The CO stripping curves of Bi/C in 1 M KOH electrolyte at a scan rate of 50 mV s^{-1}.

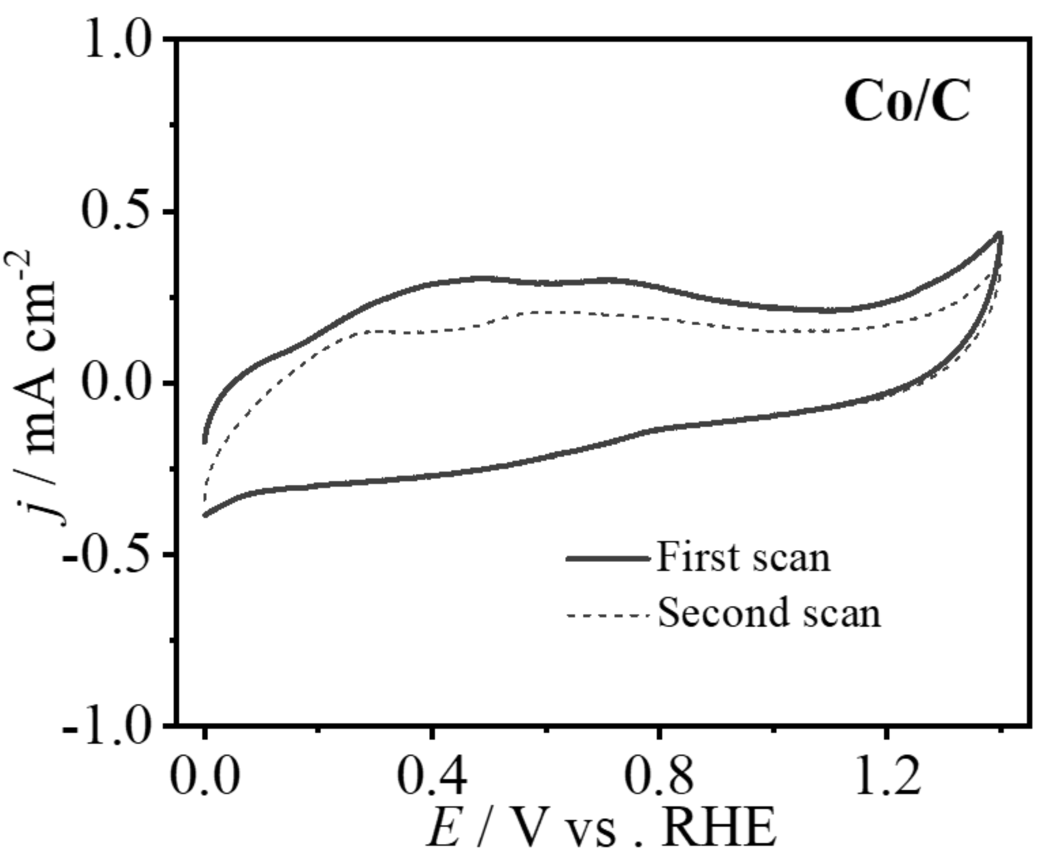

Figure S7. The CO stripping curves of Co/C in 1 M KOH electrolyte at a scan rate of 50 mV s^{-1}.

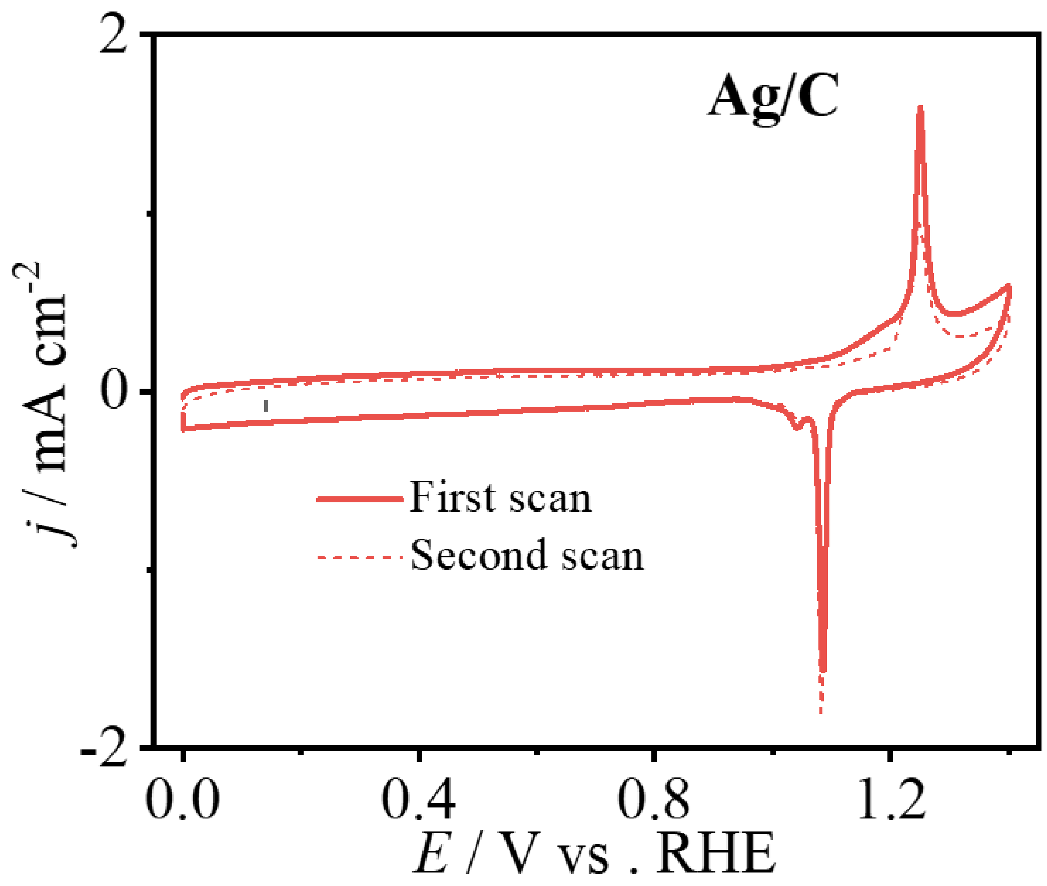

Figure S8. The CO stripping curves of Ag/C in 1 M KOH electrolyte at a scan rate of 50 mV s^{-1}.

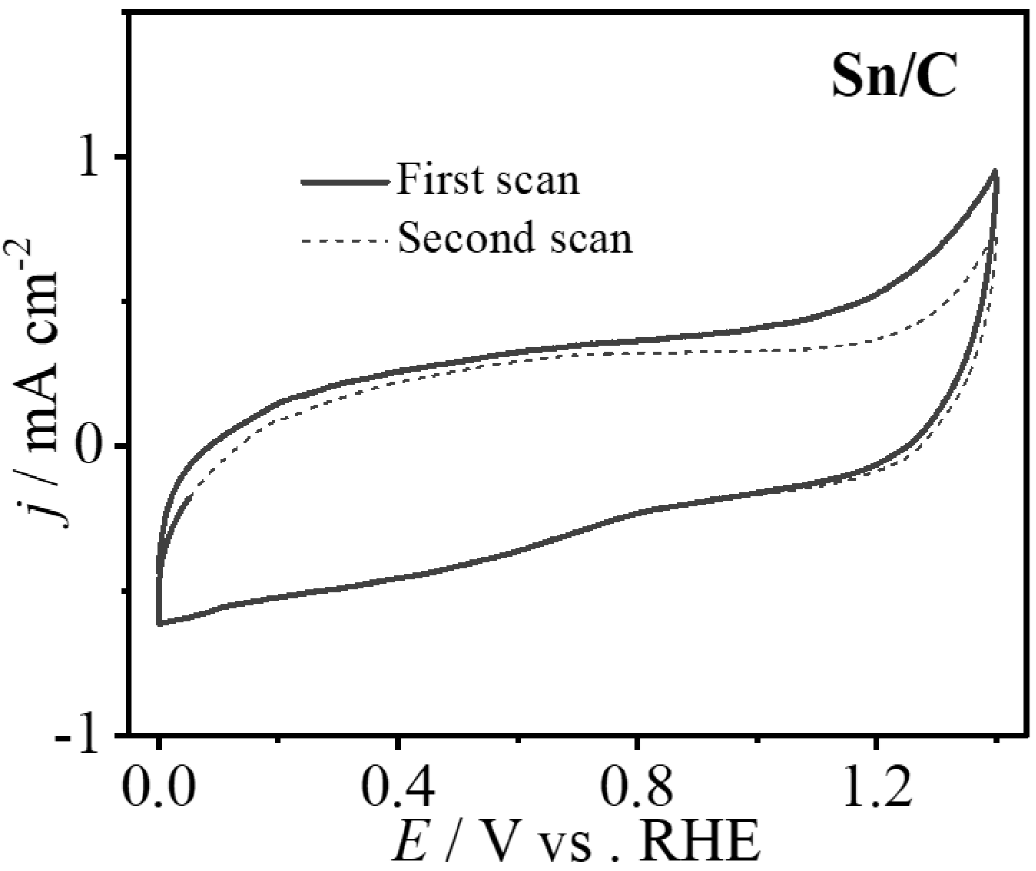

Figure S9. The CO stripping curves of Sn/C in 1 M KOH at a scan rate of 50 mV s^{-1}.

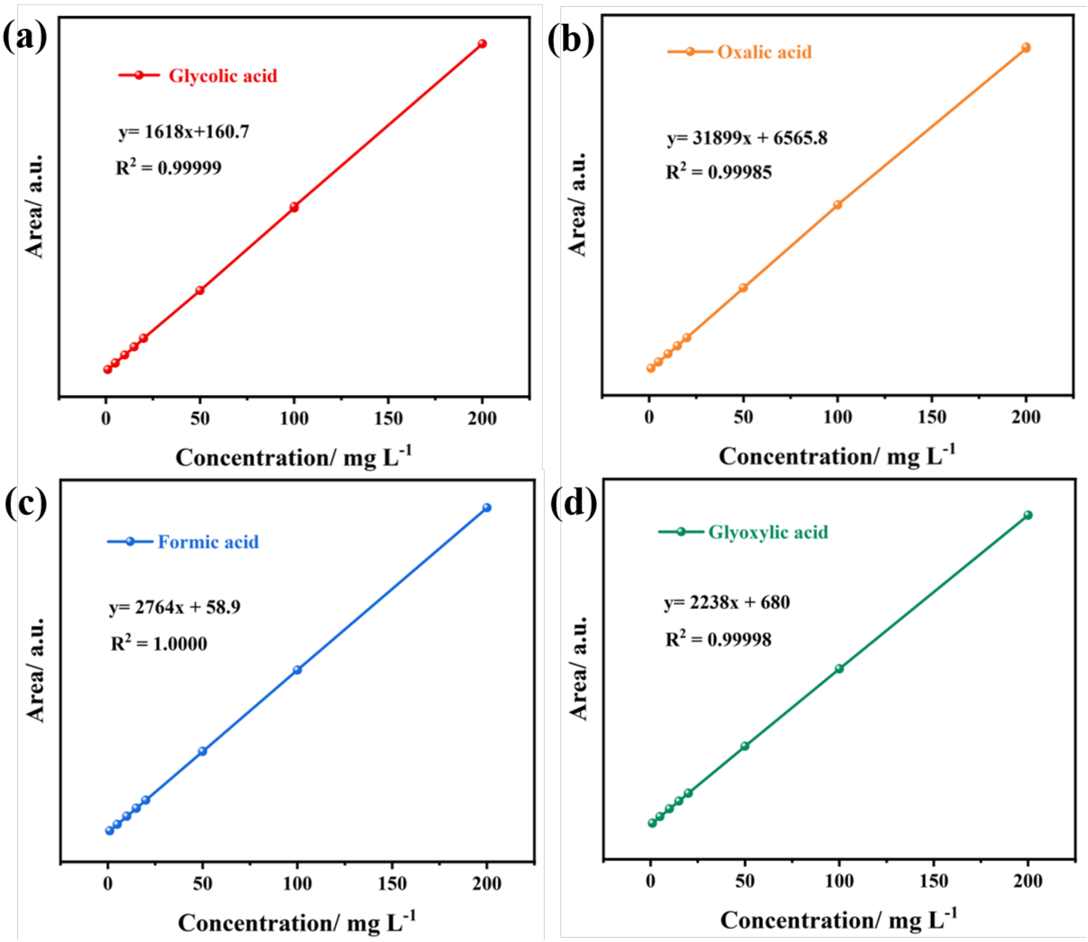

Figure S10. The high-performance liquid chromatography (HPLC) calibration curves of (a) Glycolic acid, (b) Oxalic acid, (c) Formic acid, and (d) Glyoxylic acid from 1 ppm to 200 ppm.

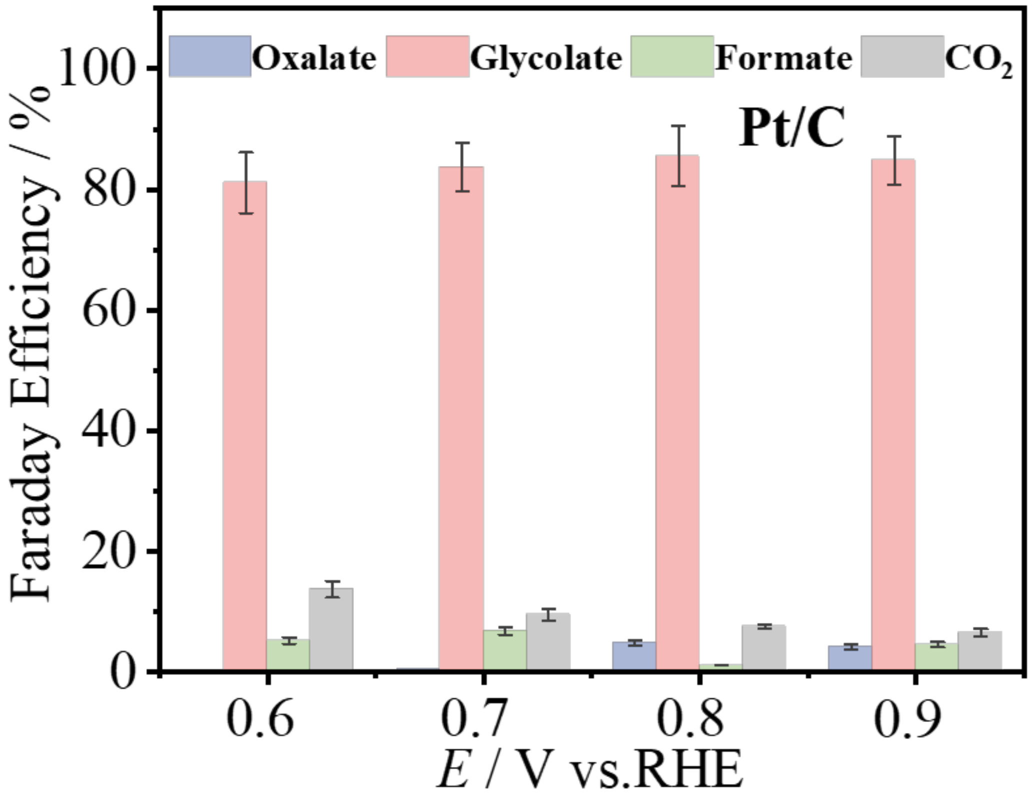

Figure S11. The Faradaic efficiency (*FE*) of various products on Pt/C in 1 M KOH and 1 M EG solution within the potential range of 0.5-0.9 V vs. RHE.
